# Delivery of a national prenatal exome sequencing service in England: a mixed methods study exploring healthcare professionals’ views and experiences

**DOI:** 10.3389/fgene.2024.1401705

**Published:** 2024-06-05

**Authors:** Michelle Peter, Rhiannon Mellis, Hannah McInnes-Dean, Morgan Daniel, Holly Walton, Jane Fisher, Kerry Leeson-Beevers, Stephanie Allen, Emma L. Baple, Ana Beleza-Meireles, Marta Bertoli, Jennifer Campbell, Natalie Canham, Deirdre Cilliers, Jan Cobben, Jacqueline Eason, Victoria Harrison, Muriel Holder-Espinasse, Alison Male, Sahar Mansour, Alec McEwan, Soo-Mi Park, Audrey Smith, Alison Stewart, Dagmar Tapon, Pradeep Vasudevan, Denise Williams, Wing Han Wu, Lyn S. Chitty, Melissa Hill

**Affiliations:** ^1^ North Thames Genomic Laboratory Hub, Great Ormond Street Hospital for Children NHS Foundation Trust, London, United Kingdom; ^2^ Genetics and Genomic Medicine, UCL Great Ormond Street Institute of Child Health, London, United Kingdom; ^3^ Antenatal Results and Choices, London, United Kingdom; ^4^ Department of Applied Health Research, University College London, London, United Kingdom; ^5^ Alström Syndrome UK, Torquay, United Kingdom; ^6^ West Midlands Regional Genetics Laboratory, Central and South Genomic Laboratory Hub, Birmingham, United Kingdom; ^7^ RILD Wellcome Wolfson Centre, University of Exeter Medical School, Royal Devon University Healthcare NHS Foundation Trust, Exeter, United Kingdom; ^8^ Peninsula Clinical Genetics Service, School, Royal Devon University Healthcare NHS Foundation Trust, Exeter, United Kingdom; ^9^ Bristol Regional Genetics Service, St Michael’s Hospital, Bristol, United Kingdom; ^10^ Northern Genetics Service, International Centre for Life, Newcastle upon Tyne, United Kingdom; ^11^ Department of Clinical Genetics, Leeds Teaching Hospitals NHS Trust, Leeds, United Kingdom; ^12^ Liverpool Centre for Genomic Medicine, Liverpool Women’s NHS Foundation Trust, Liverpool, United Kingdom; ^13^ Oxford Centre for Genomic Medicine, Oxford, United Kingdom; ^14^ Faculty of Medicine, Imperial College & North West Thames Regional Genetics Service, London, United Kingdom; ^15^ Nottingham Regional Genetics Service, City Hospital Campus, Nottingham University Hospitals NHS Trust, Nottingham, United Kingdom; ^16^ Wessex Clinical Genetics Service, Princess Anne Hospital, Southampton, United Kingdom; ^17^ Clinical Genetics Department, Guy’s Hospital, Guy’s and St Thomas’ NHS Foundation Trust, London, United Kingdom; ^18^ St George’s University Hospitals NHS Foundation Trust, London, United Kingdom; ^19^ Department of Obstetrics and Gynaecology, Nottingham University Hospitals, Nottingham, United Kingdom; ^20^ Department of Clinical Genetics, Cambridge University Hospitals NHS Foundation Trust, Cambridge, United Kingdom; ^21^ Manchester Centre for Genomic Medicine, St Mary’s Hospital, Manchester, United Kingdom; ^22^ Sheffield Clinical Genomics Service, Sheffield, United Kingdom; ^23^ Queen Charlotte’s and Chelsea Hospital, Imperial College Healthcare NHS Trust, London, United Kingdom; ^24^ Department of Clinical Genetics, University Hospitals of Leicester NHS Trust, Leicester, United Kingdom; ^25^ Clinical Genetics Unit, Birmingham Women’s and Children’s NHS Foundation Trust, Birmingham, United Kingdom

**Keywords:** prenatal exome sequencing, prenatal diagnosis, genomic sequencing, healthcare professionals, genetic services, service evaluation, genomic medicine service

## Abstract

**Introduction:**

In October 2020, rapid prenatal exome sequencing (pES) was introduced into routine National Health Service (NHS) care in England, requiring the coordination of care from specialist genetics, fetal medicine (FM) and laboratory services. This mixed methods study explored the experiences of professionals involved in delivering the pES service during the first 2 years of its delivery in the NHS.

**Methods:**

A survey (*n* = 159) and semi-structured interviews (*n* = 63) with healthcare professionals, including clinical geneticists, FM specialists, and clinical scientists (interviews only) were used to address: 1) Views on the pES service; 2) Capacity and resources involved in offering pES; 3) Awareness, knowledge, and educational needs; and 4) Ambitions and goals for the future.

**Results:**

Overall, professionals were positive about the pES service with 77% rating it as Good or Excellent. A number of benefits were reported, including the increased opportunity for receiving actionable results for parental decision-making, improving equity of access to genomic tests and fostering close relationships between FM and genetics departments. Nonetheless, there was evidence that the shift to offering pES in a clinical setting had brought some challenges, such as additional clinic time, administrative processes, perceived lack of autonomy in decision-making regarding pES eligibility and difficulty engaging with peripheral maternity units. Concerns were also raised about the lack of confidence and gaps in genomics knowledge amongst non-genetics professionals - especially midwives. However, the findings also highlighted value in both FM, obstetric and genetics professionals benefiting from further training with a focus on recognising and managing prenatally diagnosed genetic conditions.

**Conclusion:**

Healthcare professionals are enthusiastic about the benefits of pES, and through multi-collaborative working, have developed relationships that have contributed to effective communication across specialisms. Although limitations on resources and variation in knowledge about pES have impacted service delivery, professionals were hopeful that improvements to infrastructure and the upskilling of all professionals involved in the pathway would optimise the benefits of pES for both parents and professionals.

## 1 Introduction

Prenatal exome sequencing (pES) has shown promise as a powerful tool for prenatal diagnosis of fetuses with anomalies. With the potential to improve the diagnostic yield of monogenic conditions (Lord et al., 2019; Petrovski et al., 2019), pES provides parents with clinically useful information for pregnancy, early neonatal management, and longer-term prognosis (Skirton et al., 2014; Chandler et al., 2018).

**FIGURE 1:**
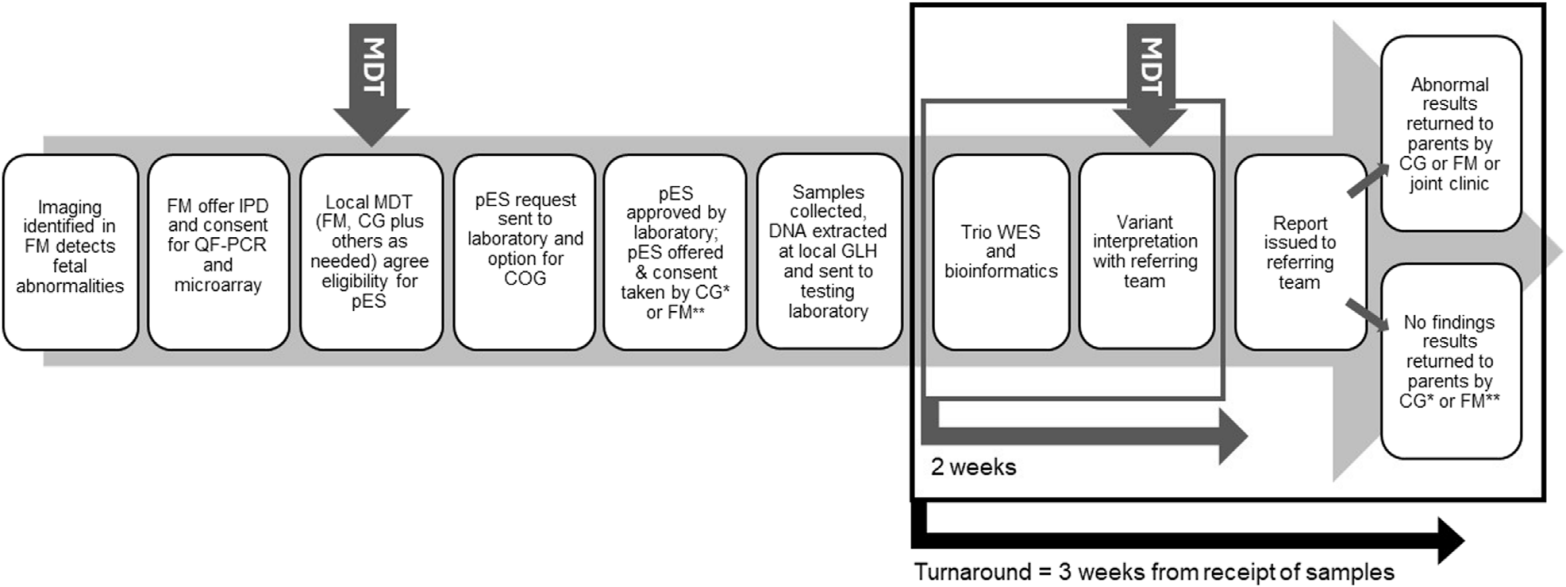
Overview of the pES pathway. Local pathways can vary in which staff groups are involved in taking consent and return of results. MDT, Multi-disciplinary team; FM, Fetal medicine; IPD, Invasive prenatal diagnosis; OF-PCR, Quantitative fluorescent-polymerase chain reaction; CG, Clinical genetics; COG, Clinical oversight group; GLH, Genomic laboratory hub; WES, Whole exome sequencing; *, may include genetic counsellors; **, may include midwives.

Recent decisions to mainstream pES in England have been guided by evidence on its feasibility and clinical utility (Chandler et al., 2018; Lord et al., 2019; Petrovski et al., 2019). Introduced into antenatal care by England’s National Health Service’s (NHS) Genomic Medicine Service (GMS) in October 2020 (NHS England and NHS Improvement, 2020), pES is now offered routinely when abnormalities detected on fetal imaging are suspected to have monogenic aetiology following multidisciplinary review that includes a clinical geneticist, and molecular diagnosis may influence parental decision-making or pregnancy or neonatal management (Figure 1 illustrates the general pES pathway). Testing ideally involves trio sequencing, and analysis uses a panel of more than 1200 genes (Genomics England, 2021) associated with congenital structural anomalies presenting prenatally or in the newborn period.

The eligibility criteria and panel approach used for analysis is designed to maximise the identification of monogenetic conditions whilst minimising incidental findings. Parents receive pre-test counselling during which the possibility of the identification of incidental findings and variants of uncertain significance (VUS) is discussed. Parents are not able to opt out of receiving incidental findings since they are detected through the process of trio sequencing to identify an underlying cause for the fetal abnormalities. However, current national guidelines advise that both VUS and incidental findings are only reported to parents after multidisciplinary team (MDT) discussion determines the variant is relevant to the fetal phenotype or will impact on parental health or future reproductive risks (Supplementary Material).

Currently offered via referral through clinical genetics, England’s pES service relies on coordination between specialist genetics and fetal medicine (FM) professionals and clinical scientists. Considerations around professional education, the need for expert counselling, and the type of results returned to families have been highlighted as key issues when introducing pES into mainstream care (Quinlan-Jones et al., 2016; Brew et al., 2019; Chandler et al., 2022; Mellis et al., 2022). Understanding the impact of delivering pES as a national service from the perspective of healthcare professionals is necessary for recognising areas of success and efficiency, highlighting areas for improvement, and identifying support needs. Optimising EXome PREnatal Sequencing Services (EXPRESS) (NIHR127829) is a national study examining the delivery of pES across England through the NHS GMS (Hill et al., 2022). In the mixed methods study described here, we have used surveys and interviews with professionals to address the research question: What does delivery of a national pES service look like from the perspective of healthcare professionals, and what should it look like in the future?

To build a picture of pES service delivery from implementation through the first 2 years of service in the NHS, we focused on four areas: 1) views on the pES service), 2) capacity and resources, 3) awareness, knowledge, and educational needs and 4) ambitions and goals for the future.

## 2 Materials and methods

### 2.1 Study design

In this mixed methods study, a quantitative survey and qualitative interviews with professionals explored their experiences of delivering a national pES service in a publicly funded national health service.

### 2.2 Setting

The NHS GMS consolidates all genomic testing into a unified service delivered through seven regional NHS Genomic Laboratory Hubs (GLHs) and NHS Genomic Medicine Service Alliances (GMSAs). A national Genomic Test Directory defines which genomic tests are available through this service. pES is listed as R21, and laboratory analysis is performed at two of the seven GLHs (NHS North Thames and NHS Central and South GLHs). Within the seven GLHs/GMSAs, there are 17 regional genomic services who work with their linked FM and maternity units to refer parents for pES. Where a referral for pES is declined by the testing laboratory, professionals may appeal the case to the national Clinical Oversight Group. The Clinical Oversight Group, set up in March 2021, includes at least one clinical geneticist from each GLH to provide independent decision-making regarding pES eligibility in “borderline” or complex cases. National figures indicate that up until March 2024, approximately 2235 referrals for pES had been made, of which around 1700 have been accepted (North Thames GLH and Central and South GLH (March 2024), Personal Communication).

### 2.3 Participants and recruitment

*Surveys* **:** Clinical leads for the R21 service from the 17 regional genomics services identified 15–18 professionals from their region who were involved in the pES pathway. These potential participants were emailed a study invitation, participant information sheet and a link to complete the survey on SurveyMonkey. Reminder emails were sent two, four, and 5 weeks after the initial invitation. The survey was open from 21 March 2022 until 4^th^ May 2022.

*Interviews*: Professionals involved in the pES pathway were identified by the research team and were emailed a study invitation and participant information sheet asking them to contact the research team if they wanted to take part. Written or audio-recorded verbal consent was obtained prior to the interview. Interviews were conducted by MP, HM, RM, and MH between November 2020 and December 2022.

### 2.4 Survey and topic guide development

*Surveys:* The survey (Supplementary Material) was developed with guidance from professionals experienced in offering pES. To answer our research questions, for this paper, we report only on the findings from survey items 3–5, 16–17, 23–24, 30, and 33–39. Findings relating to pES referral and results processes will be reported elsewhere. The closed-text items in this paper assessed demographic information, views on the pES service, the impact on administrative and clinical time as a consequence of the pES service, awareness of guidelines and policies, knowledge of the eligibility criteria (EC) for pES (Supplementary Material) and educational needs and preferences. Open-text boxes provided the opportunity for more detailed feedback. A pilot version of the survey was circulated to professionals in one GLH/GMSA (North Thames). Suggestions to improve the survey were incorporated into the final survey.

*Interviews*: The interview topic guide was first drafted by MH and revised following feedback from HM and MP (experienced qualitative researchers), and LSC, RM and DT (professionals with experiential knowledge of pES). The topic guide (Supplementary Material) explored experiences of the pES service, goals and challenges for pES service delivery, care pathways, and educational and support needs.

### 2.5 Data analysis

Survey and interview data were analysed separately and then integrated so that interview findings add context to the survey responses.

*Surveys*: Descriptive statistics using frequencies and proportions were calculated. For comparative analyses of the survey data, professionals were categorised as FM professionals or genetics professionals. Comparative analyses were conducted to identify relationships between demographic and outcome variables and to identify differences between groups. Depending on the variable type, data was either analysed as continuous or categorical. Independent *t*-tests, chi-squared associations of independence and two proportions *z*-tests were used to assess differences between groups. All analyses of quantitative data were conducted using R 4.0.2 (R Core Team, 2020).

*Interviews* **:** Interviews were audio-recorded and transcribed verbatim. All data was pseudo-anonymised prior to analysis. Analysis followed the principles of thematic analysis (Braun V, 2021), with findings generated using a team-based codebook approach (MacQueen et al., 1998). Interviews were coded against a codebook by HW, MP, MD and MH using both inductive and deductive approaches (Bradley et al., 2007). Analysis was facilitated by NVivo 13 (QSR International, Pty Ltd, Australia).

## 3 Results

Of 280 surveys distributed, 179 were started. Surveys where participants had started but entered no data (*n* = 4) or completed demographic information only (*n* = 15) were excluded. The final dataset comprises 159 surveys (response rate: 57%). Of 134 professionals invited to participate, 63 were interviewed, 70 did not respond, and one actively declined (recruitment rate: 47%). Interviews were conducted by video call (*n* = 53), face-to-face (*n* = 6) and telephone (*n* = 2) and lasted between 23 and 80 min (median duration 44 min). Participant characteristics are presented in Table 1.

**TABLE 1:** Participant characteristics for the survey and interviews.

	N	(%)		N	(%)
*Survey participants*					
**Region in England**			**Professional group**		
North West	24	15%	FM professionals	86	54%
North East and Yorkshire	24	16%	Genetics professionals	73	46%
East	25	16%			
Central and South	21	13%	**Years of experience**		
North Thames	22	14%	0–5	50	31%
South East	17	11%	6–15	55	35%
South West	24	15%	>16	54	34%
**Professional role**					
Consultant clinical geneticist (CG)†	44	28%			
Genetic counsellor†	24	15%			
Registrar/Trainee/CG Fellow†	5	3%			
FM Consultant*	51	32%			
Registrar/Trainee/FM Fellow*	4	3%			
Registrar/Trainee/Obstetric Fellow*	1	1%			
Obstetrician*	5	3%			
FM midwife*	22	14%			
Screening midwife*	2	1%			
Neonatologist*	1	1%			
	**N**	**(%)**		**N**	**(%)**
*Interview participants*					
**Region in England**			**Professional role**		
North West	8	13%	Clinical genetics professional	24	38%
North East and Yorkshire	8	13%	FM professional	21	33%
East	10	16%	FM midwife	6	10%
Central and South	11	17%	Genetic counsellor	7	11%
North Thames	12	19%	Clinical scientist	5	8%
South East	4	6%			
South West	7	11%			
NA	3	5%			

*Key:* FM, fetal medicine; CG, clinical geneticist; * = categorised as FM professional; † = categorised as genetics professional.

### 3.1 Views on the pES service

#### 3.1.1 Familiarity with the pES service

Survey respondents were asked to describe their familiarity with the pES service (Table 2). Very few reported being unfamiliar or having limited understanding of the service. Although around half (*n* = 46; 53%) of FM professionals offered pES in their clinical practice, this was significantly higher for genetics professionals (53% vs. 71%; *p* = 033). Level of familiarity was not related to years of experience (*p* = 263).

**TABLE 2:** Level of familiarity with pES (survey participants).

	N (%)		*p*-value
	FM professionals	Genetics professionals	
Familiar with pES but not used it	8 (9%)	3 (4%)	*p* = .331
Heard of pES but limited understanding	2 (2%)	0 (0%)	*p* = .550
Offer pES in my clinical practice	46 (53%)	52 (71%)	*p* = .033
Support but do not personally offer pES	30 (35%)	18 (21%)	*p* = .220

#### 3.1.2 Overall views on the pES service

The pES service was rated highly by most survey respondents. Of those with experience of using the service, 77% (*n* = 111) rated it as good or excellent, 17% (*n* = 25) as average, and 4% (*n* = 7) as poor or very poor. Ratings did not differ across the two professional groups.

Accordingly, many interview participants were positive about the pES service, describing it as “fantastic,” “brilliant” and “amazing”. Benefits for parents were expressed, most commonly how the service increases the possibility of finding a diagnosis and provides families with “more clarity” and “actionable information” to support their decision-making (Table 3, Q1). pES was felt to help parents’ decision-making around pregnancy management, delivery, and care after birth. Professionals also praised the rapid response of colleagues, and many noted that the service assisted them as professionals, helping them to offer more to parents (Table 3, Q2). Being able to offer a national service where access is not dependent on where parents live was also viewed positively (Table 3, Q3).

**TABLE 3:** Views on the pES service.

Quote number	Illustrative quote	
*Overall views on the pES service*		
Q1	“We know with R21 [pES] that we’re giving them the most detailed information that we have access to. And I think that is the biggest advantage because we have the real potential of giving a definitive diagnosis in the mid trimester usually and then we give them the options.” - Professional 13, FM Consultant	
Q2	“In the past it used to be so frustrating to sit with the parents and say we don’t know what’s wrong but there is something wrong, whereas you know we are able to say you know we have much more detailed tests…You know it feels better. I mean I feel better, to be honest.”–Professional 55, FM Consultant	
Q3	“It just means that there is more equity in accessing genetic testing across the country, so it’s not dependant on your contacts or what your lab’s able to do for you, and it feels like there’s just a much more equitable service.” - Professional 41, Clinical geneticist	
*Mixed views on EC and referral processes*		
Q4	“I can see why it [the eligibility criteria] was chosen for those things and I can understand that it isn't always going to be what it is now and it’s already been amended, hasn’t it, different things added in.” - Professional 31, Clinical geneticist	
Q5	I have to admit to some frustration sometimes when I think there’s a case where a patient would benefit from exome sequencing and it’s turned down. - Professional 63, FM Consultant	
Q6	“Our ultimate aim would be that we as clinicians, as experienced clinical genetics consultants should be the gatekeepers of the service rather than the lab and having to pass–because we feel that in trying to seek approval, valuable time is lost.” - Professional 21, Clinical geneticist	
*Scope of the pES service*		
Q7	“There’s definitely an argument for doing that because you then don’t get, you know, too much noise and it gives people more uncertainty and more trauma and more anxiety in lots of ways.So I think I’m happier with it being a panel than an exome.” - Professional 19, Clinical geneticist	
Q8	“You’ve got to expect that more genes will be added as they are discovered, then as long as there’s, you know, adequate evidence that they’re real.You can’t freeze genetic knowledge at a point in time and say if you’re unlucky enough to develop a disorder that isn't discovered until after this then tough we’re not going to identify it.” - Professional 46, Clinical geneticist	
*Good communication and multidisciplinary team working is crucial*		
Q9	“The thing that was a challenge that how is that going to go and how are we going to communicate, but I think it was very transparent and very open, so once we’ve overcome that fear of “oh it”s a new thing, how is that going to work?’, I think it wasn’t a massive issue in the end.” - Professional 54, Clinical geneticist	
Q10	“I would say this time last year before we went into the centralisation process, you know, we didn’t really know each other or have a relationship particularly but I think we’ve definitely built that over the last year, because we’re having regular weekly meetings, regular contact.” - Professional 17, Genetic counsellor	
Q11	“Switching it to Teams has been better because the attendance is better…there’s the ability for people to be there and actually we get more attendance in the neo-natal teams now as well, which is great, because some of it’s about management of cases and things as well, so it works really well.” - Professional 17, Genetic counsellor	

#### 3.1.3 Mixed views on EC and referral processes

For the seven survey respondents who rated the pES service as poor or very poor, free text comments indicated that negative views predominantly focused on the EC or the referral process, where professionals and scientists at the testing GLH review each referral. The EC was viewed as “too narrow,” and the referral process seen as resulting in the declining of “too many cases.” These views were echoed in several interviews, with many professionals wanting broader or more flexible EC. Many professionals did, however, note that the current EC was an appropriate starting point with suggestions that it would likely “change with time” as knowledge and experience increases (Table 3, Q4). Several FM and genetic professionals across the region also described the referral process as “frustrating and obstructive” (Table 3, Q5) and wanted a more autonomous approach to referrals with less “gatekeeping” from the testing GLH (Table 3, Q6). Similarly, some FM professionals wanted the autonomy to make referrals directly, with less input from local genetics teams.

#### 3.1.4 Scope of the pES service

Concerns were raised by some that using a gene panel for pES analysis could increase the potential to “miss lots of other cases” (Professional 63, FM Consultant). Others expressed disappointment that new gene discovery was curtailed. Most however, felt that a panel was appropriate for “minimising confusing information”– particularly because of the difficulties in interpreting fetal phenotypic information (Table 3, Q7). There was optimism that the panel would expand as knowledge of gene variants increases (Table 3, Q8), and that having a regular review of the panel against the evolving literature would be important (now implemented). Nearly all professionals agreed that only pathogenic and likely pathogenic findings should be reported as discussing uncertain findings with parents adds a “layer of complexity”.

#### 3.1.5 Good communication and multidisciplinary team working is crucial

Participants highlighted that service delivery is dependent on clear care pathways and effective multidisciplinary team working between FM, genetics, and laboratory teams. Whilst some had initially found the reorganisation of genetics services into a unified national GMS “very difficult” and reported having had “anxieties” about whether newly aligned clinical departments and laboratories would successfully collaborate, most professionals felt that departments were now working well together (Table 3, Q9). Communication was initially assisted where relationships were “longstanding” and where colleagues “know each other well”. For teams who had not previously worked together, the reorganisation had fostered relationships between different specialisms (Table 3, Q10). Relationships between FM and genetics were particularly strong when genetics and FM teams were co-located at the same hospital, when genetics professionals were embedded within FM units or when FM and genetics ran joint clinics. Although some practical challenges were described where the two departments were geographically far from each other, workarounds such as the use of virtual MDTs had been implemented with success (Table 3, Q11).

### 3.2 Capacity and resources

#### 3.2.1 How much administrative and clinical time does pES add?

Survey respondents were asked how much additional administrative and clinic time was involved in offering pES (Supplementary Material). FM professionals most frequently (*n* = 27; 31%) reported an increase in time spent on administrative tasks by 0–30 min [*Z* = 10.62, *p* = .001].

• Offering pES had a greater impact on administration time for genetics professionals who more frequently reported it added 31–60 min of time [*Z* = 6.96, *p* = .008]. Increases to clinic time as a result of offering pES were reported by FM and genetics professionals to the same extent.

Many interview participants also reported that delivering the pES service added significant administrative and clinical time to their workload (Table 4, Q1). The challenge of balancing an “already busy clinic” with completing paperwork was evident for both FM and genetics professionals. Feelings of frustration at the “unseen” workload that “isn’t really tariffed anywhere” (Professional 25, FM Consultant) were, however, more common amongst genetics professionals (Table 4, Q2), reflecting that the additional administrative work associated with the pES service was more commonly taken on by genetics than FM professionals. Accordingly, in regions where the role of the genetics team was to take consent and organise testing, or when one or two FM colleagues with an interest in genetics took on additional tasks, FM professionals did not see a substantial increase in their workload (Table 4, Q3). Having the resources to include additional staff in the pES service, such as genetic counsellors or midwives, who could take consent, complete paperwork, or organise samples could ease the “burden” (Table 4, Q4).

**TABLE 4:** Capacity and resources.

Quote number	Illustrative quote
*How much administrative and clinical time does pES add?*	
Q1	“I cannot tell you how pressurising that is because really to see somebody, to scan them, to counsel them about what you’ve found, to talk through options, to do an invasive test, to talk through the options of the analysis of the sample and to do that in a genuinely informed way, not rushing people, probably even with a bright couple who kind of know what they want to do from the word off, you’re talking at least an hour/hour and 20 minutes. If you’re doing it via an interpreter and you’re adding in all the forms that are required for R21, you’re talking about at least 2 hours’ work and, even then, you feel like you’ve rushed people.” - Professional 25, FM Consultant
Q2	“Yesterday it took me 25 min to see a couple–it took me another half an hour to complete the forms - I had to fill in three records of discussion, the request form and then the blood forms–seven forms–it took me just as long to fill in the forms as to see the couple.” - Professional 53, Clinical geneticist
Q3	“If I was actually doing the consenting, that would add a bit of time to patients that I was seeing. But I think that, I don’t think it’s a huge burden adding an exome.” - Professional 56, FM Consultant
Q4	“When the service first started we did the appointments jointly with the genetic counsellor which was amazing and then they just said “We can’t do it anymore because we’re too busy.I think it’s massively helpful to have a genetic counsellor to join those appointments.so at the moment no one’s helping me do any of it.” - Professional 39, Clinical geneticist

### 3.3 Awareness, knowledge, and educational needs

#### 3.3.1 Awareness of national pES systems

Most survey respondents reported knowing where to find the current EC (*n* = 132; 86%). Just over half (53%; *n* = 81) reported awareness of the online national educational multidisciplinary team (MDT) meetings and 67% (*n* = 51) of these respondents felt the meetings were extremely or very valuable (Supplementary Material). Around half of survey respondents (*n* = 75; 49%) had used the national Clinical Oversight Group, and 57% (*n* = 41) of these respondents reported that it was extremely or very valuable, whilst 19% (*n* = 14) felt it was slightly or not at all valuable.

Most genetics professionals knew where to access guidelines and the latest pES service updates. There were, however, concerns that this knowledge was inconsistent across hospitals for FM professionals (Table 5, Q1). FM and genetics professionals described the national MDT meetings as “really interesting”, a useful education resource—particularly for FM professionals, and a valuable opportunity for sharing complex cases with experts from across England (Table 5, Q2). These meetings were also an important space for professionals to share views about the service, report clinical evidence, and affect change (Table 5, Q3), such as the EC expansion to include isolated non-immune fetal hydrops. Professionals familiar with the Clinical Oversight Group described the independent arbitration as “a useful process” that was helpful for “grey area” cases that “don’t quite fit” the criteria.

**TABLE 5:** Awareness, knowledge, and educational needs.

Quote number	Illustrative quote
*Awareness of national pES systems*	
Q1	“I’m a geneticist, so I think I get it fed to me from various different resources, and we’re constantly in liaison with [the testing laboratory]. I’m not sure that other fetal medicine teams that are not the larger ones, or antenatal teams, know where to access that information.” Professional 41, Clinical geneticist
Q2	“I do think that educational MDTs about sharing interesting cases, sharing cases where there’s maybe been challenges is incredibly useful, because then you get that sharing of knowledge across the whole system, rather than only the cases that you see and I think that will be an advantage for everyone.” - Professional 29, Clinical scientist
Q3	“People presented evidence at that meeting and then it was discussed and it was agreed that we would change the criteria. And I think that’s really good, it comes across as much more democratic than, you know, a group of people saying this is what will be. And I think it gave people a forum in which to discuss those things.”–Professional 1, Clinical geneticist
*Knowledge and understanding of the pES EC*	
Q4	“Part of the reason we’ve been so careful to involve the genetics department is to avoid getting ourselves into the situation of promising the test and then having to weigh back a week later because we get told actually no this baby doesn’t meet the criteria to offer it.” - Professional 13, FM consultant
Q5	“So, there is a huge amount of variability between specialists, depending on the specialty, and which hospital they might work in, and their exposure to genomics as to how good they are at knowing about the national test directory…I think the tertiary centres, which I think are more specialist, have grasped onto those much better. But there still seems like a huge gap between different hospitals and their ability to understand mainstream genomics.” - Professional 41, Clinical geneticist
*Education and training needs to support pES service delivery*	
Q6	“I think there’s a huge amount of change that’s happened in a very short space of time and I think genetics will become part of mainstream medicine, it’s inevitable but I think a lot of the work around the education, the training of health professionals has kind of lagged behind a little bit”. - Professional 51, Genetic counsellor
Q7	“I thought it was very interesting in the year that all the NHS education [2.15] were being thrown at doctors, genomic medicine was not one of the top five priorities to teach medical students that were announced that year and that seemed to me to be a little bit bizarre.” - Professional 1, Clinical geneticist
Q8	“I don't really understand why there is a drive for these specialities to become geneticists and I don't see how as much as training and education you want to put in place, I don't see how they can get the expertise that we’ve been building on for the last 20, 30, 40 years…I think there are limitations in terms of what mainstream clinicians can do and can’t do.” - Professional 54, Clinical geneticist
Q9	Some of us have got a better handle on those issues and how to talk about them that can come up, the thing is with those issues–they’re not so different thematically to the issues that might come up with microarray–they’re all on a similar theme and so, you know, if you’re consenting people properly for doing a microarray, it’s not such a massive leap to consenting them for an R21 either. - Professional 25, FM consultant
Q10	“I would never be able to see an abnormal result and say–or I don’t think I would be, unless it’s something like a condition that’s very well known, but I’d never be able to counsel what that genetic condition is, what that means to the baby, what the options are and things like that.” - Professional 36, FM Consultant
Q11	“I think even the fetal medicine consultants are reluctant [to take consent] …I’m the midwife that does the consent for the exome…some of the consultants are not having to do it because they don't want to do it within their clinics, and they feel it’s outside of their norm. So, yeah, they rely on me.”–Professional 26, FM midwife
Q12	“We did a series of repeated Teams sessions where we not only talked about who was eligible for the test, what the test did, but how to talk to patients about it and talk about some of the results that we’d started to see coming through.But we haven’t really met with any great enthusiasm for people to take that up. And I think that comes from their lack of time, you know, and the steep learning curve that it would require and, yeah, just sort of general fear I think.” - Professional 17, Genetic counsellor
Q13	Locally I think we have a particular problem in that we have several fetal medicine units in our region and only two consultants that are specialising in prenatal genetics. So, you know, just spreading ourselves very thinly. - Professional 1, Clinical geneticist

#### 3.3.2 Knowledge and understanding of the pES EC

The mean survey score for EC knowledge was 3.99 (*SD* = 1.32, range = 1–5) which, given the maximum possible score of 5, indicates that most professionals had good knowledge. All professionals (*n* = 145; 100%) knew that multiple structural anomalies are eligible and isolated mild ventriculomegaly is not. There was less certainty around large echogenic kidneys and major CNS abnormalities, with 63% (*n* = 91) and 79% (*n* = 114) correctly selecting these options, respectively.

Interview participants felt that that more training was “extremely important” for FM professionals to understand exactly what conditions meet the pES EC (Table 5, Q4). In addition, many interview participants raised that there was wide variability in awareness of pES and understanding of the EC amongst FM professionals in peripheral hospitals (Table 5, Q5). Whilst some hospitals were felt to have good knowledge, others were felt to have “very poor understanding” or “they don’t think about it” (Professional 31, Clinical geneticist).

#### 3.3.3 Education and training needs to support pES service delivery

**FIGURE 2:**
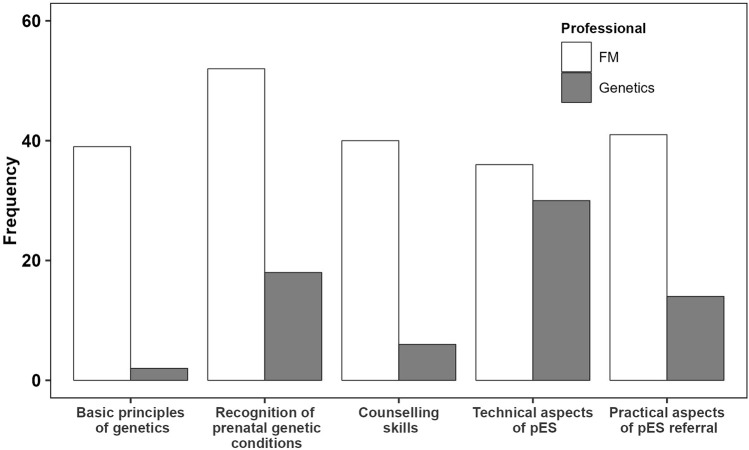
Education and training needs split by professional role.

Survey respondents reported how they kept updated about pES, whether their units had taken measures to support inclusivity and equity of access, and their preferred approach to pES training (Table 6). Respondents wanted to learn more about the recognition and management of genetic conditions in the prenatal period (*n* = 70; 53%); the technical aspects of pES and variant interpretation (*n* = 66; 50%); and the practical aspects of pES referral (*n* = 55; 42%) (Figure 2). Significantly more training needs [*t* (130) = 6.78, *p* < 001] were reported by FM (*M* = 2.8, *SD* = 1.5) than genetics professionals (*M* = 1.4, *SD* = 0.9), with more FM professionals wanting to learn basic science principles of genetics and genomics [*Z* = 36.07, *p* < 001] and counselling skills [*Z* = 27.06, *p* < 001]. Notably, although more FM professionals wanted to learn about the recognition and management of genetic conditions in the prenatal period [*Z* = 20.11, *p* < 001], this topic was also of interest to just over a quarter of genetics professionals (*n* = 18; 26%). FM and genetics professionals were equally keen to learn the technical aspects of pES and variant interpretation.

**TABLE 6:** Information about current training materials and preferences for training methods.

Ways updated about pES	N (%)	Training preference	N (%)
NHSE/I webinars	79 (53%)	Webinars	122 (82%)
Professional body webinars	31 (21%)	In person training	57 (38%)
Local training	74 (50%)	Online training course	74 (50%)
Journal articles	45 (30%)	Written information	62 (42%)
Conference talks	77 (52%)	Mandatory training	6 (4%)
Guidelines	46 (31%)	Don’t know	9 (6%)
Laboratory website	27 (18%)		
Measures to support inclusivity/equity of access			
Cultural competency training	74 (48%)		
Departmental policy in place	46 (30%)		
Interpreters and advocates	134 (88%)		
Multi-language parent information	31 (20%)		

*Key*: NHSE/I = NHS England and NHS Improvement

Many interview participants felt that greater education about pES was needed to optimise the service, especially for FM doctors and midwives (Table 5, Q6). There were also broader concerns raised about the lack of genomics in current medical school curricula (Table 5, Q7). Some participants did, however, feel that FM professionals should not be expected to become genomics specialists (Table 5, Q8).

Confidence of FM doctors to consent parents and return results varied. Some spoke confidently, viewing pES as analogous to more familiar genetic tests, such as microarray (Table 5, Q9). Others, however, described feeling “anxious” about counselling parents and wanted more training. FM doctors also worried about returning results for uncommon conditions or uncertain findings (Table 5, Q10). The confidence and involvement of FM midwives in delivering pES varied widely. Where a FM midwife had a special interest in genomics, their knowledge and counselling skills were integral to the delivery of the pES service (Table 5, Q11). The more common view, however, was that FM midwives often find “the whole thing quite intimidating” (Professional 25, FM Consultant).

Several genetics professionals described conducting local workshops and training events to raise awareness about the pES service, with the wider aim of educating FM teams. Training sessions, however, were not always well-attended. This was attributed to time pressures, a fear of genomics or not seeing pES as part of their role (Table 5, Q12). The need for further education for genetics professionals was also highlighted, with some noting that they would benefit from learning more about conditions specific to the prenatal period. In addition, both FM and genetics professionals explained that it was sometimes challenging for FM teams to access clinical geneticists who were prenatal specialists (Table 5, Q13).

### 3.4 Ambitions and goals for the future

**FIGURE 3:**
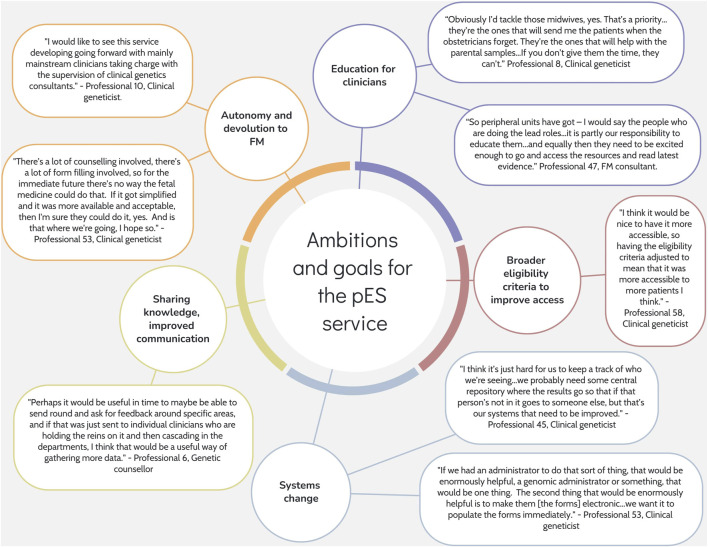
Ambitions and goals for the pES service.

Interview participants were asked about their ambitions for the pES service and suggestions for improvement (Figure 3). Most suggestions related to education and training for FM professionals, with a particular focus on targeting midwives as “a priority”. Professionals envisioned a service where there was more trust and autonomy in their decision-making as specialists. Many professionals viewed the involvement of genetics professionals as integral to pES service delivery but felt that the service would eventually devolve to FM with genetics oversight. Professionals also anticipated that the EC would widen to improve access. There were suggestions to increase the availability of patient information in different formats and languages. In addition, professionals were hopeful for improved communication and case sharing across the service as well as upgrades to pathways, IT systems, and additional staffing to manage the increased workload.

## 4 Discussion

This study is the first to offer insight into professionals’ experiences of delivering a national pES service at the outset of implementation, and their views on what it should look like in the future. We found that both FM and genetics professionals were positive about England’s pES service. In line with other research (Quinlan-Jones et al., 2016; Narayanan et al., 2018; Mellis et al., 2022) professionals were enthusiastic about the clinical benefits of pES and described how the increased opportunity to receive actionable results during a critical period for parental decision-making had helped them to improve support for parents. Furthermore, and in keeping with NHS England’s vision to provide equity of access to genomic medicine (NHS England, 2022) embedding pES into mainstream care was seen as fundamental for ensuring that all parents eligible for pES will be offered it.

In recent work exploring stakeholder views of moving pES from research into a clinical setting in the NHS, professionals anticipated that effective service delivery would need FM and genetics professionals to work together (Mellis et al., 2022). The benefits of collaborative working for variant interpretation when offering pES have also been reported. Our findings indicate that implementing the pES service has fostered close relationships between FM and genetics teams and provided opportunities for learning and knowledge exchange across specialisms that supports successful service delivery.

However, offering pES routinely in a clinical setting has brought several challenges. Additional administrative processes and extra clinic time needed to counsel parents were experienced by professionals from all disciplines, with genetic professionals impacted to a greater extent. Although many professionals welcomed the reassurance of discussing cases with specialists, others reported a perceived lack of autonomy in decision-making that left them frustrated at having to seek approval for referrals despite their own expertise. System-level changes to the referral process may come with time, however, improvements to existing resources, streamlining of care pathways and the inclusion of additional staff groups to support pES delivery could be addressed immediately. For example, expanding the genomics workforce to include staff such as “genomics associates” who can support the consent process and administrative tasks could provide professionals with more time for specialist tasks and allow the pES service to be delivered more efficiently. Improvements to IT systems could also facilitate the various processes involved in sharing documentation with the labs and monitoring test status. Similar approaches to improve efficiency have been suggested in recent research looking at offering whole genome sequencing in the NHS GMS (Friedrich et al., 2023).

Some professionals questioned the use of a restricted panel of genes for pES analysis, preferring a more agnostic approach to testing that would optimise the clinical potential of pES. It was, however, acknowledged that widening the gene panel for pES would increase the chance of detecting VUS which, given the limitations in the systems used to classify variants (Lord et al., 2019), could place unmanageable demand on the service. Detection of VUS is not unique to pES: uncertain findings also arise through use of chromosomal microarray (CMA) (Wapner et al., 2012; Hillman et al., 2013) and our findings echo long-standing worries about the anxiety caused to parents by the return of uncertain results from prenatal testing (Westerfield et al., 2014; Jez et al., 2015; Harding et al., 2020; Lewis et al., 2021). In this regard, a future ambition for many professionals was the sharing of data on public genetic repositories. The use of databases such as DECIPHER, will be key as new gene variants are discovered and better understanding of existing variants evolves (Lord et al., 2019). An important consideration already highlighted will be determining responsibility for re-contacting parents when new knowledge emerges (Horn and Parker, 2018).

Previous work has shown the value of clinicians with a good understanding of pES providing detailed counselling that makes clear the limitations and potential implications of the test (Best et al., 2018; Mellis et al., 2022). The importance of accurate fetal phenotyping when offering pES has also been discussed (Aarabi et al., 2018; Monaghan et al., 2020). The current study identified gaps in genomics knowledge amongst FM professionals and a need for more genetics professionals with an understanding of prenatally diagnosed conditions. Worries about returning results from pES were also noted. As such, a key ambition for the pES service, highlighted by many professionals, will be improved access to education. This could help FM doctors and midwives improve their genomics knowledge and confidence in counselling for pES, for instance how to support parents when VUS results and incidental findings have been identified. Training would also help improve knowledge of prenatally identified conditions amongst genetics specialists new to working in the prenatal setting.

The multiple benefits of upskilling midwives to support the pES service was highlighted by many participants in our study. As has been observed elsewhere in the NHS nursing and midwifery workforce (Carpenter-Clawson et al., 2023), midwives feel limited by their knowledge and confidence about genomics despite recognising its importance in patient care. Further, and in line with recent work (Seed et al., 2022) professionals in this study highlighted a need for the teaching of genomics to begin at undergraduate level in order to better equip the medical professionals of the future. Educational opportunities are available to support the mainstreaming of genomics. For instance, the Genomics Education Programme (Genomics Education Programme, 2022), delivered by Health Education England, offers online access to short courses and clinical resources. Training materials that integrate case examples could also be effective; this approach has been shown to increase understanding of genetic concepts (Hajek et al., 2022) and aligns with the experiences of the professionals in this study who valued the national MDTs where pES cases were discussed. In addition, training facilitated by a mentor could be an effective way of establishing meaningful engagement (Bishop et al., 2019), and secondment to specialist teams for experiential learning might help to better embed knowledge and support clinical practice (McClaren et al., 2020). A common concern highlighted in this study was that professionals lack the time for further education. It is vital that protected time for additional learning is given to all professionals involved in the pES pathway. Moreover, for professionals working in local, non-specialist maternity units where awareness of the pES service may be limited, further education must be considered a priority to ensure an equitable service.

An important caveat is that whilst the introduction of the national pES service is new, the questions raised by the professionals in this study regarding implementation of a new healthcare service are not. Similar issues have been encountered elsewhere. For instance, the impact of limited knowledge of prenatal phenotypes on variant interpretation, reservations about the preparedness of clinicians, and concerns about how to counsel parents about uncertain results have been reported in studies exploring professional views on the introduction of CMA into prenatal settings (Robson et al., 2017; Lewis et al., 2021). Our findings should, therefore, be interpreted in light of these broader issues associated with the implementation of large-scale services that require adaptation within a local context.

## 5 Strengths and limitations

A strength of this study is that the views of professionals from a range of backgrounds and across all regions of England were included. In addition, both quantitative and qualitative information has guided our understanding of pES service delivery. Our study commenced at the same time as the pES service was launched, allowing us the unique opportunity to capture experiences during the first years of implementation. A limitation of the study is that interviews were conducted over a 2-year period, thus individual experiences may have differed over time as the service became more established, particularly as the service was first implemented when COVID restrictions were in place. A further limitation is that respondents were self-selecting and there may be bias towards those with strong views about the pES service.

## 6 Conclusion

Professionals working in FM and genetics settings are enthusiastic about the benefits of pES for parents and welcome the introduction of a national pES service in England. Collaborative working between FM and genetics teams has been central to the successful delivery of pES. Our findings highlight that further education and training is needed and that constraints on time and resources can impact the ability to deliver the pES service efficiently. Improvements to IT and staffing along with a sustained effort to upskill both FM and genetics professionals could optimise the benefits of pES and lead to improved experiences for both parents and professionals.

## Data Availability

The raw data supporting the conclusion of this article will be made available by the authors, without undue reservation.
