# Supplementary material for: Delivery of a national prenatal exome sequencing service in England: a mixed methods study exploring healthcare professionals’ views and experiences
Source: Front Genet. 2024 Jun 5;15:1401705. doi: 10.3389/fgene.2024.1401705 (PMC11188373; doi:10.3389/fgene.2024.1401705)
Supplement: Supplementary file 1 [file DataSheet2.PDF]

Supplementary materials

Topic guide

Background

1. Can you start by telling me a little about your professional background and your current role?

Views on the GMS

2. What are your thoughts on the new Genomic Medicine Service and the plan to embed genomic medicine in the NHS?
    a. What do you think about the reconfiguration of the genetic services into hubs?

3. Thinking about the GMS as a whole, what do you see as the main opportunities and benefits the GMS will provide now and in the future?

4. At the outset, what did you anticipate the main challenges to incorporating genomics into mainstream NHS practice might be and what challenges are we still facing a year into implementation?
    b. Are there general ways these challenges be addressed? Prompts: *leadership, national guidance funding model, clinician time, support for clinicians to attend from clinical managers*

Setting up the prenatal ES service

5. Why do you think rapid prenatal ES was selected as one of the first tests to offer in the GMS?
    a. What do you see as the main benefits of offering rapid prenatal ES in the NHS?
    b. What are the challenges or limitations of using rapid pES in the NHS? Practical/logistical/ethical etc?

6. Prior to the launch of the service had you been involved in discussions or planning for the overall prenatal ES service?

7. Do you know of any plans to audit or monitor the service to determine if implementation has been successful?

8. What do you think have been the main challenges of setting up a national prenatal ES service and what challenges are we still facing a year into implementation?
    a. Has anything been smoother than expected?
    b. What new problems do you anticipate going forward?
    c. Do you anticipate any differences in how individual GLH's will deliver the service?

9. What have been the main challenges of setting up the prenatal ES service at a **local** level and what challenges are we still facing a year into implementation? Have you seen any barriers?
    a. Prompt; engagement of local FMUs, cultural barriers in local populations

10. What are your thoughts about raising awareness of the service and how we should be training health professionals that might be expected to offer WES?
    a. Is any education and training of professionals being delivered nationally?
    b. Is any education and training occurring at a local level?

11. Do you find the NHSE-led educational MDTs useful?
    c. Did you attend any of these?

12. Do you know if referrals/queries are being received from peripheral units in your GLH?
    a. If so, how do you think this has been achieved?
    b. If not, what do you think needs to be done to increase awareness?

13. How prepared to offer ES are the fetal medicine professionals in your region?
    a. Are links between fetal medicine and genetics and the lab well established?
    b. How have genetics professionals been supporting their fetal medicine colleagues as the service is getting established?
    c. Can you give examples of what is working well or which areas of communication need improvement?

Current processes for offering pES

14. Can you describe the practical aspects of organising these tests and how the care pathways for the service work in your GLH/GMSA right now?
    a. How is it decided who will be offered rapid exome sequencing?
    d. Do genetics and FMU run a joint clinic?
    e. Is pES mentioned to patients before contacting the lab?
    f. What do you tell parents about the turnaround time for receiving results?
    g. Who consents patients? Face to face or virtual?
    b. Who returns results?
    c. Which professionals are involved in this process? (MDT meetings?)
    d. How do MDT decisions work, could the MDT process be improved?
    e. Have there been any issues in getting samples to the lab or communication with the lab?

15. Have there been any changes to care pathways, MDTs or other processes over the past year (in your GMSA or more widely) that have made delivering the R21 service more or less efficient?

16. In March 2021, the Clinical Oversight Group was developed to support the decision-making process regarding eligibility criteria for complex cases. Has the implementation of this group been useful? Maybe

17. Has being able to offer ES made a change to your own clinical practice?
    a. Can you give examples of how?

18. What impact (if any) has the service had on your time?
    h. Prompt – time spent in appointments / time spent doing admin / time spent interpreting variants / organising MDTs etc.

19. In your experience, how have parents responded to being offered rapid ES?
    a. What do you think are the most important points to get across during pre-test counselling?

Prompt: types of results such as no result or uncertain results / incidental findings / limitations of the technology?

    b. How long does the discussion tend to last?
    c. Do you think the parents are able to make an "informed decision"?
    d. Do you have any thoughts about the information that has been developed for parents?
    e. What can we do to better support parents? Is there a need for more / different / other formats (online) information? Website/app/video?
    f. Do you anticipate any issues with equity of access in your region? Barriers to accessing ES for particular groups in your local community?
    g. Are there any strategies to address equity of access and inclusivity?

20. From your experience, what are some of the reasons that people decline prenatal exome sequencing?

21. Are these the same or different to reasons to decline established prenatal genetic testing?

22. What is the process for results interpretation and returning results?
    a. How do the clinicians interact with the lab when results are returned? Do they support result interpretation?Is there a standard process in place for decision making around variant classification?
    b. How do MDT decisions work, could the MDT process be improved?
    c. Are MDTs used at time of referral AND for interpreting results?Can you tell me about your experience when giving results to patients? - Diagnosis / No primary findings / Uncertain findings
    d. Do you have any thoughts on the **timing** of results and decisions about termination?

Thoughts on current policy and guidelines

23. Do you feel there is sufficient/clear enough guidance for health professionals on using ES for prenatal diagnosis?
    a. What (if any) further guidance would you find useful?

24. Were the national monthly meetings an effective way to discuss and effect changes in the service?
    a. Are there are there other benefits to these meetings?
    b. Are there disadvantages to these meetings?
    c. Other than these meetings are there ways of having your voice heard about the R21 service?

25. Do you have any thoughts on the approach of offering ES by referral through the genetics team?
    a. Looking to the future, do you think this test will continue to be offered by referral through the genetics team or will this ultimately shift to FMUs?

26. What do you think of the current eligibility criteria?
    a. Do you think the criteria will continue to change and evolve over time?

27. What do you think of the current approach to analysis where the scientists only look at a restricted panel of genes?
    a. Do you think this will change over time?

28. What are your views on reporting only pathogenic and likely pathogenic findings? Do you have any thoughts on whether/how incidental findings, variants of uncertain significance, variable penetrance should be reported?
    a. What is it like to discuss these types of results with parents?
    b. How well do parents cope with these types of findings?

29. Looked for additional findings, such as cancer susceptibility genes, are not offered in the current R21 service; do you think there is a place for offering this in pregnancy? (for the baby? For the parents?)
    a. Prompt: benefits/ concerns/ what guidance should we follow – ACMG guidelines?
    b. Do you think offering secondary findings would influence decisions about termination of pregnancy?

30. Is there anything you would like to change about the service as it develops and what are your ambitions for the service over the next six months?

Prompts: review of eligibility criteria / routine process for re-analysis

Other issues

31. What do you think about offering ES as a non-invasive test?

32. Are there any ethical issues specific to prenatal ES that require consideration?
