# Supplementary material for: Delivery of a national prenatal exome sequencing service in England: a mixed methods study exploring healthcare professionals’ views and experiences
Source: Front Genet. 2024 Jun 5;15:1401705. doi: 10.3389/fgene.2024.1401705 (PMC11188373; doi:10.3389/fgene.2024.1401705)
Supplement: Supplementary file 2 [file DataSheet4.DOCX]

**Supplementary materials**

**Link to the NHS R21 Guidance document – available online**

The NHS Guidance document outlines the eligibility criteria and referral process for the R21 pES service.

It can be found online at: <https://norththamesgenomics.nhs.uk/wp-content/uploads/2023/08/Rapid-Exome-Sequencing-Guidance.pdf>

Accessed on 18/04/2024
