# Supplementary material for: Delivery of a national prenatal exome sequencing service in England: a mixed methods study exploring healthcare professionals’ views and experiences
Source: Front Genet. 2024 Jun 5;15:1401705. doi: 10.3389/fgene.2024.1401705 (PMC11188373; doi:10.3389/fgene.2024.1401705)
Supplement: Supplementary file 3 [file Table1.DOCX]

**Supplementary materials**

**Table 1.** Additional administrative and clinic time because of pES.

|  | **N (%)** | | **p-value** |
| --- | --- | --- | --- |
|  |  |  |  |
|  | **FM professionals** | **Genetics professionals** |  |
| *Administrative time* |  |  |  |
| 0-30 mins | 27 (31%) | 14 (19%) | *p* = .001 |
| 31-60 mins | 16 (19%) | 37 (51%) | *p* = .008 |
| > 60 mins | 8 (9%) | 13 (18%) | *p* = .693 |
| Don't know | 11 (13%) | 4 (13%) | - |
| Not applicable | 24 (28%) | 5 (28%) | - |
|  |  |  |  |
| *Clinic time* |  |  |  |
| 0-30 mins | 40 (47%) | 37 (51%) | *p* = .699 |
| 31-60 mins | 16 (19%) | 13 (18%) | *p* = .669 |
| > 60 mins | 3 (4%) | 9 (12%) | *p* = .128 |
| Don't know | 7 (8%) | 8 (11%) | - |
| Not applicable | 20 (23%) | 6 (11%) | - |
|  |  |  |  |
| *Note:* Those reporting "Don't know" or "Not applicable" were excluded from comparative analyses with proportions re-calculated only on included data | | | |
