# Supplementary material for: Delivery of a national prenatal exome sequencing service in England: a mixed methods study exploring healthcare professionals’ views and experiences
Source: Front Genet. 2024 Jun 5;15:1401705. doi: 10.3389/fgene.2024.1401705 (PMC11188373; doi:10.3389/fgene.2024.1401705)
Supplement: Supplementary file 4 [file DataSheet3.PDF]

Supplementary materials

Scoring for knowledge about the pES EC

To assess knowledge of the indications for pES, respondents were asked to select up to five of the following clinical presentations they believed are included in the national eligibility criteria (EC) for pES: 1) multiple structural anomalies (in at least two body systems), 2) suspected skeletal dysplasia, 3) large echogenic kidneys with a normal bladder, 4) isolated mild ventriculomegaly, and 5) major CNS abnormalities (excluding neural tube defects).

Responses 1), 2), 3) and 5) are stated in the criteria whilst response 4) is not. Participants scored one for each correct answer; scores on this question could range from 0-5, with higher scores indicating greater knowledge of the pES EC.
