# Supplementary material for: Delivery of a national prenatal exome sequencing service in England: a mixed methods study exploring healthcare professionals’ views and experiences
Source: Front Genet. 2024 Jun 5;15:1401705. doi: 10.3389/fgene.2024.1401705 (PMC11188373; doi:10.3389/fgene.2024.1401705)
Supplement: Supplementary file 5 [file Table2.DOCX]

**Supplementary materials**

**Table 2.** Awareness and perceived value of the Clinical oversight group and monthly national educational MDTs

|  |  |  |
| --- | --- | --- |
|  | **N (%)** | |
|  | **Clinical oversight group** | **National educational MDT** |
| *Awareness* |  |  |
| Yes | 75 (49%) | 81 (53%) |
| No | 33 (22%) | 72 (47%) |
| Don't know | 45 (29%) | - |
|  |  |  |
| *Value* |  |  |
| Extremely valuable | 16 (15%) | 12 (16%) |
| Very valuable | 47 (45%) | 39 (51%) |
| Moderately valuable | 23 (22%) | 18 (23%) |
| Slightly valuable | 12 (12%) | 7 (9%) |
| Not at all valuable | 6 (6%) | 1 (1%) |
|  |  |  |
| *Note*: those reporting "Don't know" or "Not applicable" were excluded from comparative analyses with proportions re-calculated only on included data | | |
