# Supplementary material for: Delivery of a national prenatal exome sequencing service in England: a mixed methods study exploring healthcare professionals’ views and experiences
Source: Front Genet. 2024 Jun 5;15:1401705. doi: 10.3389/fgene.2024.1401705 (PMC11188373; doi:10.3389/fgene.2024.1401705)
Supplement: Supplementary file 6 [file DataSheet1.PDF]

EXPRESS
Optimising EXome PREnatal Sequencing Services

# Health professional survey on the NHS prenatal exome sequencing (R21) service

## 1. About the study

• This survey is part of the EXPRESS study and is intended for professionals from a range of backgrounds working in maternity, fetal medicine and genetics.

• The survey should take 15-20 minutes to complete depending on your role and experience.

• The aim of the survey is to help us understand your views on prenatal exome sequencing, how this test is delivered day-to-day in your units, what education resources are available and what else might be needed.

• You do not need to have experience or knowledge of the service to take part.

• To maintain the anonymity of participants, the survey findings will be grouped for analysis and reporting.

• If you have any queries about the survey please get in touch with the EXPRESS research team (Email: express@gosh.nhs.uk).

• If you would like to be sent a summary of the results of the survey, you can add your email address on the last page of the survey. Your email address will be kept separately from survey answers.

* 1. Please confirm that you have read and understood the participant information (sent via email with your survey invitation) and that you consent to taking part in this survey.

◯ Yes

◯ No

EXPRESS
Optimising EXome PREnatal Sequencing Services

# Health professional survey on the NHS prenatal exome sequencing (R21) service

## 2. About you

* 2. Which NHS Trust do you work for?

* 3. What is your professional role?

- ◯ Consultant Obstetrician
- ◯ Registrar/Trainee/Fellow in Obstetrics
- ◯ Consultant in Fetal Medicine
- ◯ Registrar/Sub-specialist Trainee/Fellow in Fetal Medicine
- ◯ Fetal Medicine Midwife
- ◯ Screening Midwife
- ◯ Consultant Clinical Geneticist
- ◯ Registrar/Trainee/Fellow in Clinical Genetics
- ◯ Genetic Counsellor
- ◯ Other (please specify)

* 4. How long have you been working in this role?

- ◯ < 1 year
- ◯ 1-5 years
- ◯ 6-10 years
- ◯ 11-15 years
- ◯ 16-20 years
- ◯ >20 years

# Health professional survey on the NHS prenatal exome sequencing (R21) service

## 3. About the referral and results processes and pathways

* 5. What is your overall familiarity with prenatal exome sequencing (ES)?

- ◯ I offer prenatal ES in my clinical practice
- ◯ I support women who are offered prenatal ES in my clinical practice (but I do not personally offer it)
- ◯ I am familiar with the concept of prenatal ES but I haven't used/encountered it yet in my own practice
- ◯ Other (please specify)
- ◯ I have heard of prenatal ES but I have limited understanding
- ◯ I have never heard of prenatal ES

* 6. Does your unit currently offer prenatal ES to parents?

- ◯ Yes
- ◯ No
- ◯ No, but we do refer parents to another unit where prenatal ES is offered
- ◯ I don't know

* 7. On average how many prenatal ES tests are requested by your unit each month?

- ◯ 0-1
- ◯ 2-3
- ◯ 4-5
- ◯ >5
- ◯ I don't know
- ◯ Not applicable, our unit does not offer prenatal ES

8. **Fetal Medicine Professionals only:** Which of the following best describes how you would usually access prenatal ES testing for parents attending your unit?

- ◯ We refer parents to a tertiary fetal medicine unit and prenatal ES is organised from there
- ◯ We refer parents to a genetics clinic and prenatal ES is organised from there
- ◯ We discuss the test request with a local clinical geneticist but organise prenatal ES from our unit
- ◯ We discuss the test request directly with the testing laboratory and organise prenatal ES from our unit
- ◯ Other (please specify)
- ◯ I don't know
- ◯ Not applicable, our unit does not offer prenatal ES
- ◯ Not applicable, I am not a fetal medicine professional

* 9. Who usually introduces and describes the option of prenatal ES to the parent(s) in your unit (Select all that apply)

- ☐ Clinical geneticist (Consultant/Registrar/Fellow)
- ☐ Genetic counsellor
- ☐ Obstetrician (Consultant/Registrar/Fellow)
- ☐ Fetal medicine specialist (Consultant/Registrar/Fellow)
- ☐ Other (please specify)
- ☐ Fetal medicine midwife
- ☐ Screening midwife
- ☐ I don't know
- ☐ Not applicable, our unit does not offer prenatal ES

* 10. Who usually consents the parent(s) when they have chosen to have prenatal ES? (Select all that apply)

- [ ] Clinical geneticist (Consultant/Registrar/Fellow)
- [ ] Genetic counsellor
- [ ] Genomics practitioner / genetics associate
- [ ] Obstetrician (Consultant/Registrar/Fellow)
- [ ] Fetal medicine specialist (Consultant/Registrar/Fellow)
- [ ] Other (please specify)
- [ ] Fetal medicine midwife
- [ ] Screening midwife
- [ ] I don't know
- [ ] Not applicable, our unit does not offer prenatal ES

* 11. Who usually gives the results of prenatal ES to parent(s)? (Select all that apply)

- [ ] Clinical geneticist (Consultant/Registrar/Fellow)
- [ ] Genetic counsellor
- [ ] Obstetrician (Consultant/Registrar/Fellow)
- [ ] Fetal medicine specialist (Consultant/Registrar/Fellow)
- [ ] Other (please specify)
- [ ] Fetal medicine midwife
- [ ] Screening midwife
- [ ] I don't know
- [ ] Not applicable, our unit does not offer prenatal ES

* 12. If you are using prenatal ES in your clinical practice, to what extent are genetic counsellors involved in service delivery? (Select all that apply)

- [ ] Genetic counsellors are involved in discussing ES with parents before they are consented for ES
- [ ] Genetic counsellors are involved in consenting parents for ES
- [ ] Genetic counsellors are involved in returning results to parents
- [ ] Genetic counsellors are involved in following up parents after results have been returned
- [ ] Genetic counsellors have no involvement in delivering prenatal ES in my unit
- [ ] Not applicable, our unit does not offer prenatal ES
- [ ] I don't know
- [ ] Other (please specify)

* 13. If you are using prenatal ES in your clinical practice, do you have additional staff to support service delivery within your team (e.g. genomics practitioners or genetics associates)? (Select all that apply)

- [ ] Yes, genomic practitioners / genetics associates are involved in consenting parents for ES
- [ ] Yes, genomics practitioners / genetics associates are involved in organising the collection, sending and tracking of clinical samples
- [ ] Yes, genomics practitioners / genetics associates are involved in administrative tasks such as completing forms and updating patient databases
- [ ] No, genomics practitioners / genetics associates have no involvement in delivering prenatal ES in my unit
- [ ] Not applicable, our unit does not offer prenatal ES
- [ ] I don't know
- [ ] Other (please specify)

* 14. Is the care pathway for your unit reflected in the responses to the questions above? If NO, please provide details to explain the pathway for your unit (e.g., from case identification to follow up)

- ◯ Yes
- ◯ I don't know
- ◯ No (please specify)

15. **Genetics professionals only:** If you are using prenatal ES in your clinical practice, on average, how many additional patient appointments in genetics are needed per case?

- ◯ 0
- ◯ 1
- ◯ 2
- ◯ >2
- ◯ Not applicable, I am not a genetics professional
- ◯ I don't know

* 16. If you are using prenatal ES in your clinical practice, how much additional clinical time (e.g. time spent counselling about prenatal ES specifically) is needed in existing appointments per case?

- ◯ It takes no additional time
- ◯ 0-15 minutes
- ◯ 16-30minutes
- ◯ 31-45 minutes
- ◯ 45-60 minutes
- ◯ >60 minutes
- ◯ Not applicable, I am not involved in clinical tasks for prenatal ES
- ◯ Not applicable, I do not use prenatal ES in my clinical practice
- ◯ I don't know

* 17. If you are using prenatal ES in your clinical practice, how much additional administrative time (e.g. liaising with the lab, keeping track of samples, paperwork) does this need, per case?

- ◯ It takes no additional time
- ◯ 0-15 minutes
- ◯ 16-30minutes
- ◯ 31-45 minutes
- ◯ 45-60 minutes
- ◯ >60 minutes
- ◯ Not applicable, I am not involved in administrative tasks for prenatal ES
- ◯ Not applicable, I do not use prenatal ES in my clinical practice
- ◯ I don't know

* 18. Has your team applied for or secured any additional funding (e.g. via an NHS transformation fund) to support prenatal ES service delivery in your unit (e.g. for extra staff or for extra resources)?

- ◯ Yes
- ◯ No
- ◯ Not applicable, I do not use prenatal ES in my clinical practice
- ◯ I don't know
- ◯ If you answered YES, can you please explain how you will use this funding and any plans for evaluating the impact of the funding?

EXPRESS
Optimising EXome PREnatal Sequencing Services

# Health professional survey on the NHS prenatal exome sequencing (R21) service

## 4. Discussions with parents

* 19. Have you used the record of discussion form to take consent from parents for prenatal ES?
'

◯ Yes

◯ No

◯ I don't know

* 20. What are your thoughts about the length of the record of discussion form?

◯ Too short

◯ About the right length

◯ Too long

◯ Not applicable, I have not used the record of discussion to take consent

Further comments

* 21. If you discuss prenatal ES with parents, do you use any information resources designed for patients in your discussion, such as leaflets?

◯ Yes

◯ No

◯ Not applicable, I do not personally discuss prenatal ES with parents

* 22. If you do use a leaflet, where is it from?

- ◯ NHS testing laboratory
- ◯ Own hospital trust
- ◯ Independent patient support organisation
- ◯ I don't know
- ◯ Not applicable, I don't use a leaflet
- ◯ Other organisation (please specify)

EXPRESS
Optimising EXome PREnatal Sequencing Services

# Health professional survey on the NHS prenatal exome sequencing (R21) service

## 5. Referral to the testing laboratory and eligibility criteria

* 23. Do you know where to find the eligibility criteria for prenatal ES?

- ◯ Yes
- ◯ No
- ◯ If Yes, please give details

* 24. To the best of your knowledge which of the following fetal clinical presentations are included current in the eligibility criteria for prenatal ES? (Select all that apply)

- ☐ Multiple structural anomalies (in at least two body systems)
- ☐ Suspected skeletal dysplasia
- ☐ Large echogenic kidneys with a normal bladder
- ☐ Major CNS abnormalities (excluding neural tube defects)
- ☐ Multiple contractures (excluding isolated bilateral talipes)
- ☐ Nuchal translucency of greater than 6.5mm plus another anomaly
- ☐ Non-immune fetal hydrops
- ☐ I don't know

* 25. How are you notified about any updates or changes to the eligibility criteria? (Select all that apply)

- ☐ Emails from NHSE/I
- ☐ Emails from the testing laboratory
- ☐ National training sessions or webinars
- ☐ Local training updates by clinical geneticist/genetic counsellor
- ☐ Word of mouth from colleagues
- ☐ I am not aware of any changes since I have been using the service
- ☐ Other (please specify)

* 26. In your experience, how long does it usually take from identifying a pregnancy potentially eligible for prenatal ES to having the test approved by the testing laboratory?

- ◯ Same day
- ◯ 1-2 working days
- ◯ >2 working days
- ◯ I don't know

* 27. In your experience, how long does it usually take (on average) from sending the samples for prenatal ES to getting results back?

- ◯ <7 days
- ◯ 7-9 days
- ◯ 10-12 days
- ◯ 13-15 days
- ◯ 16-18 days
- ◯ 19-21 days
- ◯ >21 days
- ◯ I don’t know

* 28. Is there anything you are aware of that has slowed down or delayed the testing process for your parent(s)? (select all that apply)

- [ ] Contacting local geneticist
- [ ] Discussion with testing laboratory
- [ ] Obtaining blood samples from parents
- [ ] Obtaining consent from parents
- [ ] Other (please describe)
- [ ] Transportation of samples to testing lab/samples getting lost in transit
- [ ] Technical issues at the testing laboratory
- [ ] Technical issues at our local laboratory

* 29. How would you rate the ease and quality of communication with the testing laboratory (e.g. regarding samples, forms, discussion of preliminary and final results)?

- ◯ Excellent
- ◯ Good
- ◯ Average
- ◯ Poor
- ◯ Very poor
- ◯ Not applicable / I don't have any communication with the lab in my role

Further comments

* 30. Are you aware that the prenatal ES service has a clinical oversight group for the purpose of decision-making around complex cases?

- ◯ Yes
- ◯ No

* 31. Have any of your cases been discussed by the clinical oversight group?

- ◯ Yes
- ◯ No
- ◯ I don't know

* 32. Please tell us your thoughts on the impact of the clinical oversight group:

The introduction of the clinical oversight group for decision-making around complex cases has been:

- Not at all valuable
- Slightly valuable
- Moderately valuable
- Very valuable
- Extremely valuable
- Not applicable/ I was not aware that the prenatal exome sequencing service has a clinical oversight group

Further comments

* 33. Are you aware that the prenatal exome sequencing service runs Educational MDTs for the purposes of discussing topics related to the service?

- Yes
- No

* 34. Please tell us your thoughts on the impact of the educational MDT:

The inclusion of the Educational MDTs to the prenatal exome sequencing service has been:

- ◯ Not at all valuable
- ◯ Slightly valuable
- ◯ Moderately valuable
- ◯ Very valuable
- ◯ Extremely valuable
- ◯ Not applicable/ I was not aware that the prenatal exome sequencing service runs Educational MDTs

Further comments

EXPRESS
Optimising EXome PREnatal Sequencing Services

# Health professional survey on the NHS prenatal exome sequencing (R21) service

## 6. Equity of access to the service

* 35. Does your unit have any of the following measures to support inclusivity and equity of access to prenatal ES? (Select all that apply)

- [ ] Cultural competency training for staff
- [ ] Departmental policy in place
- [ ] Use of interpreters and advocates
- [ ] Parent information resources in different formats / languages
- [ ] Other (please describe)

EXPRESS
Optimising EXome PREnatal Sequencing Services

# Health professional survey on the NHS prenatal exome sequencing (R21) service

## 7. Overall assessment of the service

* 36. How would you rate your overall experience of the prenatal ES service to date?

- ◯ Excellent
- ◯ Good
- ◯ Average
- ◯ Poor
- ◯ Very poor
- ◯ I have not had any experience using the service so far

EXPRESS
Optimising EXome PREnatal Sequencing Services

# Health professional survey on the NHS prenatal exome sequencing (R21) service

## 8. Health professional education

* 37. How do you keep updated about prenatal ES? (Select all that apply)

- [ ] Webinars and/or face-to-face training sessions delivered by the testing laboratory or NHSE/I
- [ ] Webinars/face-to-face training from Royal College or other professional body
- [ ] Training from local geneticist/genetic counsellor
- [ ] Journal articles
- [ ] Other (please specify)
- [ ] Talks given at conferences
- [ ] Guidelines from professional bodies
- [ ] Testing laboratory website
- [ ] I don't currently keep updated about prenatal ES

* 38. Would you like to know more about any of the following topics to support the delivery of prenatal ES in your unit? (Select all that apply)

- [ ] Basic science principles of genetics and genomics
- [ ] Recognition and management of genetic conditions in the prenatal period
- [ ] Counselling skills
- [ ] Technical aspects of prenatal ES and laboratory interpretation
- [ ] Practical aspects of the prenatal ES referral process (contacts, request forms, sending samples etc.)
- [ ] I don't know
- [ ] Other topic (please describe)

* 39. How would your educational needs best be met? (Select all that apply)

- [ ] Webinar
- [ ] Written information
- [ ] Face-to-face training (where pandemic restrictions permit)
- [ ] Inclusion in mandatory training at your hospital
- [ ] Self-paced e-learning course/module
- [ ] I don't know
- [ ] Other (please specify)

40. Finally, do you have any comments or recommendations to share about how to improve the prenatal ES service or best support staff delivering the service?

EXPRESS
Optimising EXome PREnatal Sequencing Services

# Health professional survey on the NHS prenatal exome sequencing (R21) service

## 9. Staying in touch

Please let us know if we can contact you in the future.

* 41. I would like to be sent a summary of the research findings

If Yes, please fill in your contact details in the contact box below

◯ Yes

◯ No

* 42. I would be willing to be contacted by the research team to take part in an interview about the prenatal ES service.

If Yes, please fill in your contact details in the contact box below

◯ Yes

◯ No

43. If you ticked yes to either query please put your contact details here:

Name ____________________

Email Address ____________________

Thank you for taking the time to complete our survey. If you have any questions, please get in touch with the EXPRESS team - express@gosh.nhs.uk
